# 867. Total Parenteral Nutrition in Burn Patients: A Single-center Retrospective Case Series

**DOI:** 10.1093/jbcr/irag033.337

**Published:** 2026-04-06

**Authors:** Sadie E Larsen, Lacey Patton, Alexandra Halevi, Cameron Gibson, Frederick Endorf, Arek J Wiktor

**Affiliations:** Burn & Frostbite Center at UCHealth, Englewood, CO; Burn & Frostbite Center at UCHealth, Aurora, CO; University of Colorado School of Medicine, Aurora, CO; University of Colorado, Aurora, CO; Burn & Frostbite Center at UCHealth, Aurora, CO; University of Colorado, Aurora, CO

## Abstract

**Introduction:**

Optimal nutrition is a critical component of burn care, essential for mitigating hypermetabolism, promoting wound healing, and improving outcomes. Total parenteral nutrition (TPN) is reserved for patients unable to meet their nutritional needs enterally, but its use has been associated with increased morbidity, prolonged hospitalization, and higher mortality. There is a paucity of evidence regarding the utilization of TPN in burn patients. Optimal nutrition is a critical component of burn care, essential for mitigating hypermetabolism, promoting wound healing, and improving outcomes. Total parenteral nutrition (TPN) is reserved for patients unable to meet their nutritional needs enterally, but its use has been associated with increased morbidity, prolonged hospitalization, and higher mortality. There is a paucity of evidence regarding the utilization of TPN in burn patients.

**Methods:**

We conducted a retrospective case series of burn patients requiring TPN at an ABA-verified burn center between January 1, 2015, and September 6, 2025. Data collected included demographics, burn characteristics, indications for TPN, duration, length of stay (LOS), weight trends, complications, and mortality. Quartile distribution of TPN initiation was also assessed.

**Results:**

Among 4959 burn admissions, 19 patients (0.4%) required TPN. Most were male (89.5%), average age 49.2 years [17–84]. Mean TBSA was 36.2% [4.51–85] with a mean of 27.8% 3rd-degree burns [0.5–76.5]; three had inhalation injuries. Baseline metabolic comorbidities were uncommon; only one patient had diabetes mellitus, and one had ESRD.

Indications for TPN included ileus n = X (47.4%, n = secondary to shock), Ogilvie’s syndrome (n = 2), bowel obstruction (n = 2), gastroparesis (n = 2), gastrointestinal bleed (n = X), high vasopressor requirement (n = X), Mallory-Weiss tear (n = X), and *C. difficile* colitis (n = X).

Mean time to TPN initiation was hospital day 19.7 [3–107], mean duration 9.4 days [1–24]. Eight patients required TPN in the 1st quartile of hospitalization, eight in the 2nd, three in the 3rd, and one in the 4th.

Average LOS was 55.3 days [12–127]. Discharge weight averaged 94.2% of admission [52.7%–138.4%]. Bacteremia occurred in 5 patients (26.3%). Mortality was 36.8% (7/19).

**Conclusions:**

TPN was used in a small but complex subset of burn patients, most often for ileus, frequently early in their hospitalization. Weight trends were inconsistent. Further studies are needed to clarify the risk factors and outcomes of TPN in this population.

**Applicability of Research to Practice:**

Although TPN use is rare in burn patients, it remains a critical adjunct for those with contraindications to enteral feeding, most often within the first month of hospitalization. Awareness of its early necessity, associated complications, and its potential impact on outcomes should inform multidisciplinary decision-making and nutritional planning in burn care.

**Funding for the study:**

N/A.

